# Concurrent acute myeloid leukemia and T lymphoblastic lymphoma in a patient with rearranged *PDGFRB *genes

**DOI:** 10.1186/1746-1596-7-19

**Published:** 2012-02-22

**Authors:** Hung Chang, Wen-Yu Chuang, Chien-Feng Sun, Marc R Barnard

**Affiliations:** 1 School of Medicine, Chang Gung University, Taoyuan, Taiwan; 2 Division of Hematology-Oncology Chang Gung Memorial Hospital, 5 Fu-Shing Street, Kweishan, Taoyuan 333, Taiwan; 3 Department of Pathology, Chang Gung Memorial Hospital, Taipei, Taiwan; 4 Core Flow Cytometry Laboratory, Department of Medicine, University of Massachusetts Medical School, Worcester, MA, USA

**Keywords:** Acute myeloid leukemia, T lymphoblastic lymphoma, *PDGFR *genes, Chromosomal translocation, Transplantation

## Abstract

Concurrent hematologic malignancies are relatively rare. We encountered a case of concurrent acute myeloid leukemia (AML) and T lymphoblastic lymphoma. The bone marrow chromosome analysis showed the karyotype 46, XY, t(5;12)(q33;p13), which indicated presence of *PDGFRB *gene translocations. Therefore, this disease belongs to the new WHO category of myeloid and lymphoid neoplasms with abnormalities in *PDGFRA, PDGFRB *and *FGFR1 *genes. Although such genetic mutations are prone to multi-lineage differentiation, the present case is in fact the first report of concurrent AML and T lymphoblastic lymphoma involving *PDGFRB *mutations. The patient was treated with cytarabine and daunomycin in combination with high dose dexamethasone. Allogeneic stem cell transplantation was performed after successful remission induction for both entities. The patient eventually died of chronic graft-versus-host-disease related infection. Based on such an experience, we suggest the decision of stem cell transplantation should be weighed carefully against the risks, especially when tyrosine kinase inhibitors are safe and potentially effective in dealing with such entities.

## Background

In 2008, a new class of hematopoietic system disorders, myeloid and lymphoid neoplasms with eosinophilia and abnormalities in *PDGFRA, PDGFRB *and *FGFR1 *genes, was created by WHO [1]. Although most cases presented with myeloproliferative neoplasm (MPN) and eosinophilia, this group of diseases was known to develop into multiple lineages of hematologic malignancies both in animal models and human cases [2]. In humans, concurrent myeloid neoplasms and T lymphoblastic lymphoma have been reported with *FIP1L1-PDGFRA *fusion genes [3,4] but not with any *PDGFRB *rearranged genes. Neoplastic ells bearing such mutations are susceptible to tyrosine kinase inhibitors and successful treatment experiences have been reported [5-9].

## Case presentation

**Figure 1:**
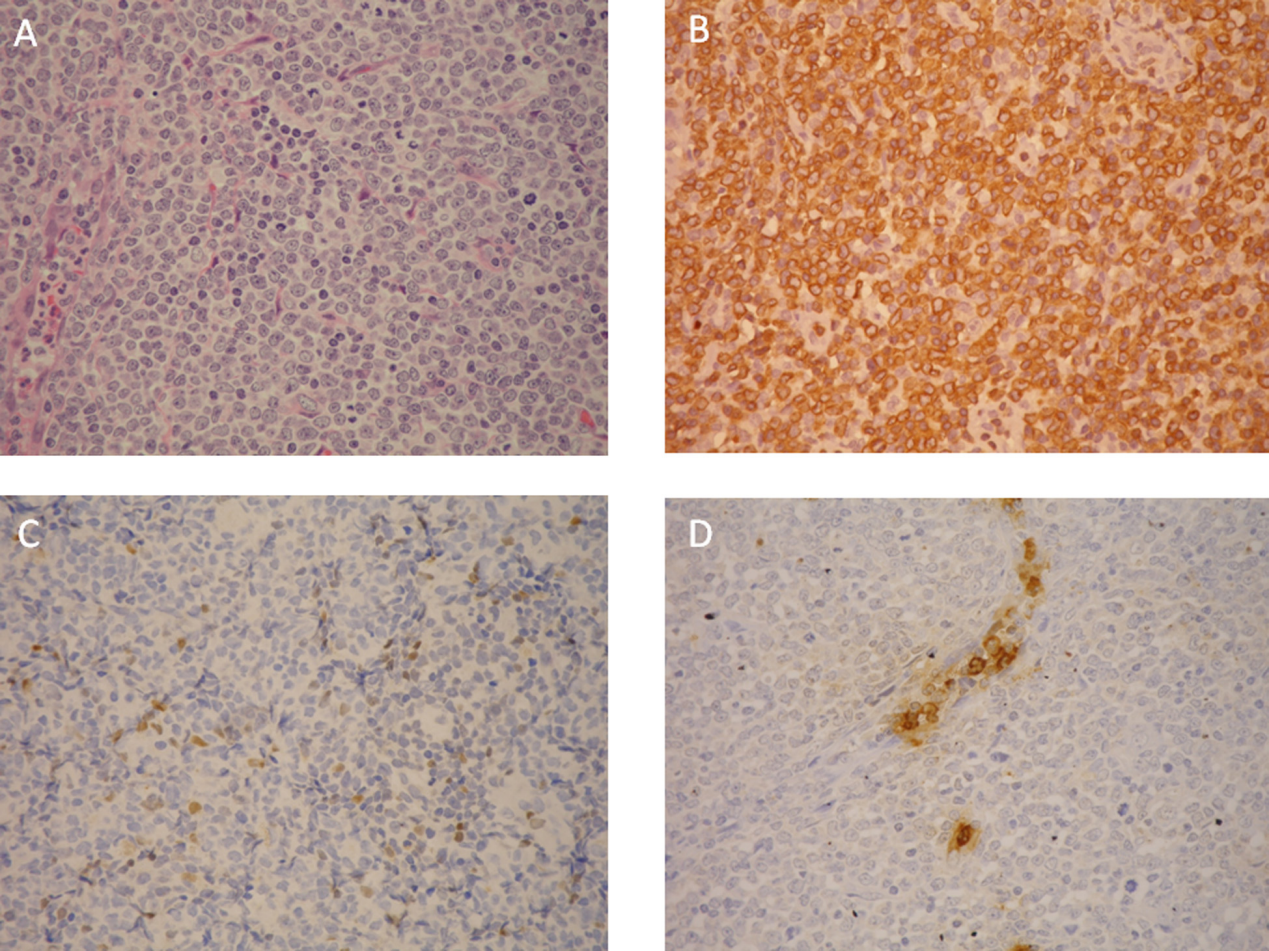
**Histology of the lymph node biopsy. A**. diffuse infiltrates of medium-sized lymphoid cells, which had fine chromatin and small nucleoli. **B**. tumor cells positive for CD3(Polyclonal). **C**.a part of tumor cells positive for TdT. **D**. tumor cells were negative for myeloperoxidase(Polyclonal).

A 41-year-old businessman presented with multiple subcutaneous nodules in the arms and trunk 3 weeks before admission. These nodules were rapidly enlarging but neither painful nor pruritic. He also reported several systemic symptoms including malaise, drenching night sweat and generalized arthralgia. His urine amount decreased markedly and he gained 3 kilograms before admission. At admission, subcutaneous nodules were found in his arms, trunk and thighs. Enlarged lymph nodes that were fixed, discrete and elastic were palpated in neck, axillary, and inguinal areas. Liver and spleen were not palpable. Marked general edema was observed. Other physical findings were unremarkable. A complete blood count revealed a hemoglobin level 10.9 g/L, a platelet count 53 × 10^9^/L, a white cell count 70.7 × 10^9^/L with 20% blasts, 3% promyelocytes, 7.5% myelocytes, 3% metamyelocytes, 31% neutrophils, 0.5% eosinopils, 24.3% monocytes and 10.8% lymphocytes. The liver biochemistry profiles were normal but the renal function tests showed creatinine 2.21 mg/dL, uric acid 15.5 mg/dL, lactate dehydrogenase 612 U/L, calcium 9.1 mg/dL, and phosphate 2.4 mg/dL. Under the impression of spontaneous tumor lysis syndrome, he was treated with hydration and rasburicase 0.15 mg/kilogram. His renal function and edema improved gradually. Meanwhile, a biopsy of the right inguinal lymph node was done. The sections showed fragments of lymphoid tissue with diffuse infiltrates of medium-sized lymphoid cells, which had fine chromatin and small nucleoli (Figure 1A). The tumor cells were positive for CD3 (Figure 1B), CD5 and TIA-1. A part of them were also positive for TdT (Figure 1C). They were negative for CD20, cyclin D1, myeloperoxidase (Figure 1D), CD4, CD8, CD30, CD56 and CD25. About 95% of the tumor cells were positive Ki-67. It was classified as T lymphoblastic lymphoma.

**Figure 2:**
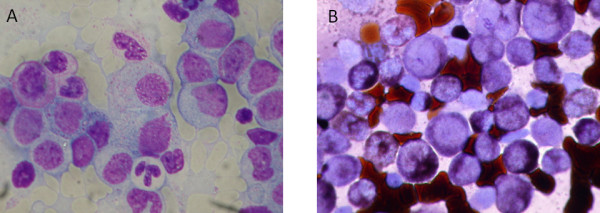
**Bone marrow aspiration smear. A**. myeloblasts accounting for 36.7% of nucleated cells. **B**. all leukemic cells positive for peroxidase.

The bone marrow aspiration smear and biopsy revealed hypercellular bone marrow with myeloblasts accounting for 36.7% of nucleated cells (Figure 2A). All leukemic cells were positive for peroxidase (Figure 2B). A flow cytometric analysis revealed the leukemic cells were positive for CD13, CD33, CD14, CD15, CD65 and negative for CD34, CD117, CD56, CD7 and CD19. In addition, the immunohistochemical stain of the bone marrow tissue section showed the blast cells were strongly positive for myeloperoxidase but negative for TdT, CD10, CD2, CD5 and CD20. The diagnosis of AML was established. The chromosome analysis revealed the karyotype 46, XY, t(5;12)(q33;p13). There was no detectable *AML1-ETO, CBFb-MYH11*, or *MLL-PTD *fusion transcripts shown by reverse transcriptase polymerase chain reactions. A fluorescent in situ hybridization (FISH) analysis with dual color *PDGFRB *probes was performed on patient's BM sample. It was positive for *PDGFRB *translocations.

He received induction chemotherapy with cytarabine (100 mg/m^2^/day continuous infusion for 7 days) and daunomycin (50/m^2^/day for 3 days). Dexamethasone 20 mg/day in divided doses was given in the same time, tapered off in a month. He had complete regression of lymph nodes and skin lesions and complete remission of AML. He subsequently received 2 cycles of postremission chemotherapy (cycle 1: cytarabine 3 g/m^2 ^for 6 doses; cycle 2: cytarabine 1 g/m^2 ^for 6 doses and etoposide 100 mg/m^2^/day for 3 days). In the state of continued remission, he underwent allogeneic stem cell transplantation from an HLA-matched sibling donor. Eleven months after transplantation, he died of chronic graft versus host disease (GVHD) and related pulmonary infection.

## Discussion

Concurrent myeloid and lymphoid malignancies are quite uncommon. The difficulty of diagnosis can be illustrated in the present case. At presentation, generalized subcutaneous tumors and lymphadenopathies led to the diagnosis of a high grade lymphoma. The pathological diagnosis of T lymphoblastic lymphoma was made unequivocally by typical morphology and detailed immunophenotypical studies. The clinical course was complicated with spontaneous tumor lysis syndrome, which typically occurred in high grade lymphoma and therefore supported the diagnosis of T lymphoblastic lymphoma. Although lymphoblastic lymphoma may involve bone marrow or peripheral blood, the results of bone marrow studies revealed the leukemic cells did not belong to the T-lineage. The strongly positive myeloperoxidase staining suggested the diagnosis of AML. This was further confirmed definitively by its typical immunophenotypical profile. Some leukemic cells of AML may occasionally express T cell markers. In our case, however, the leukemia cells was found negative for CD2, CD5 and CD7, making aberrant expression of AML relatively unlikely. The diagnosis of concomitant AML and T lymphoblastic lymphoma, was confirmed.

Although it appears unusual, such concurrence is not coincidental. In English literature, similar cases have been reported. In 2007, Metzgerot et al. reported 7 cases of AML or T lymphoblastic lymphoma bearing *FIP1L1-PDGFA *fusion genes. In 2 cases, T lymphoblastic lymphoma was found synchronously with myeloid neoplasms (acute eosinophilic leukemia in one and MPN in the other) [4]. In 2008, Capovilla et al. reported another case of synchronous chronic eosinophilic leukemia and T lymphoblastic lymphoma. Like the previous report, the leukemic cells were found to bear *FIP1L1-PDGFRA *fusion genes [3]. It appears from these reports that such genetic aberrations may lead to mixed lineage differentiation. In contrast to the previous reports with *PDGFRA *gene rearrangements, the present case was found to bear t(5;12)(q33;p13). This translocation most likely involves *PDGFRB *genes mapped on chromosome 5p. This assumption was further confirmed by studies of FISH. Based on chromosomal mapping and prior literature reports, the most likely partner gene is *ETV6 *[10].

In 2008 WHO classification, a new class of hematopoietic diseases, myeloid and lymphoid neoplasms with eosinophilia and abnormalities in *PDGFRA, PDGFRB *and *FGFR1 *genes, were introduced [1]. In such a classification system, all cases of concurrent myeloid neoplasms and T lymphomblastic lymphoma, including the present case, belong to the same category. While most cases of this entity presented with MPNs, the genetic aberrations have a wide spectrum of differentiations, including eosinophilia, myelodysplastic syndrome, chronic myelomonocytic leukemia, AML, B or T cell acute lymphoblastic leukemia, T lymphoblastic lymphoma, and disorders with mast cell proliferation [2]. Most cases with *PDGFRA *gene rearrangements manifest as MPNs, usually chronic eosinophilic leukemia. *PDGFRB *gene rearrangement cases typically present as MPNs which are almost always accompanied with eosinophilia. Aberrations of *FGFR1 *genes are also commonly associated with MPNs with eosinophilia. In particular, this genetic mutation is often associated with differentiation into both myeloid and lymphoid lineages. The most common lymphoid neoplasm is T lymphoblastic lymphoma. The concurrence of myeloid and lymphoid malignancies was reported both in human cases and animal models [2].

While these reports revealed it is not uncommon for *PDGFRA *and *FGFR1 *genetically rearranged cells to differentiate into lymphoid neoplasms, hematologic diseases bearing *PDGFRB *mutations are rare [2,5]. The two largest series reported in literature included 22 and 9 cases respectively. *PDGFRB *rearranged cells are known to develop into B and T lineage lymphoma [2]. This differentiation pattern was well described in transgenic mice models [11,12]. However, according to an extensive review by Holroyd et al, patients with *PDGFRB *mutations have not been known to develop lymphoid malignancies. This is somewhat surprising and may be explained by the rarity of such mutations among humans [2]. As a result, the case in the present article is the first case report of lymphoma and concurrent AML bearing *PDGFRB *gene mutations.

Treatment of concurrent AML and T lymphoblastic lymphoma and AML is a clinical challenge. There is mounting evidence that *PDGFRA, PDGFRB *and *FGFR1 *entities respond well to tyrosine kinase inhibitors [2,5,9,10,13]. However, tyrosine kinase inhibitors are expensive and still off-label for lymphoma. Therefore, we did not consider tyrosine kinase inhibitors as the frontline treatment in this case. In view of the simultaneous expression of myeloid and lymphoid lineage features, consideration of chemotherapeutic regimens is similar to that of bi-phenotypic leukemia. Bi-phenotypic leukemia is an uncommon disorder. No consensus protocol is available for treatment. Some studies suggest that bi-phenotypic leukemia has good responses to treatment directed against AML [14-16]. As a result, we chose treatment that was based on cytarabine and daunomycin. Steroids were included in the induction phase and complete remission was achieved for both AML and lymphoma. The patient subsequently underwent sibling-matched, allogeneic stem cell transplantation, which was quite successful until the development of chronic GVHD. Although the patient died of GVHD and related infections, he remained free from both malignancies for at least 11 months after transplantation. We believe allogeneic stem cell transplantation is an effective, albeit risky treatment option. Clinicians should weigh the risk against potential benefits before deciding treatment strategies, especially when tyrosine kinase inhibitors have become relatively safe and effective options.

## Consent

Some of the laboratory and genetic testing included in this study was done by surplus clinical samples. An informed consent to utilize the samples was available for review. The consent form was approved by the institution review board in Chang Gung Memorial Hospital.

## Competing interests

The authors declare that they have no competing interests.

## Authors' contributions

HC wrote this manuscript. WYC and CFS conducted the pathology interpretation and drafted the related parts in the article. MRB revised the article. All authors read and approved the final manuscript.
